# Effect of Aminating Lignin Loading with Arbuscular Mycorrhizal Fungi on Soil Aggregate Structure Improvement

**DOI:** 10.3390/polym16121701

**Published:** 2024-06-14

**Authors:** Chenghui Hu, Tingting Xu, Shumei Wang, Huiyang Bian, Hongqi Dai

**Affiliations:** Jiangsu Co-Innovation Center of Efficient Processing and Utilization of Forest Resources, Nanjing Forestry University, Nanjing 210037, China; hch@njfu.edu.cn (C.H.); salyting@njfu.edu.cn (T.X.); wsm@njfu.edu.cn (S.W.); hybian1992@njfu.edu.cn (H.B.)

**Keywords:** aminated lignin, load arbuscular mycorrhizal fungi, soil aggregate structure, improvement, plant growth

## Abstract

Lignin is an important component of plant fiber raw materials, and is a three-dimensional network structure aromatic polymer with abundant resources and a complex structure in nature. Lignin is generally used as industrial waste, and its potential value has not been fully utilized. Modern agriculture extensively uses chemical fertilizers, leading to the gradual degradation of soil fertility and structure, which seriously affects crop growth, nutrient transport, and root respiration function. Based on soil bulk density, porosity, aggregates, and their stability indicators, this study analyzed the effects of aminated industrial lignin and its loading with arbuscular mycorrhizal fungi on soil structure improvement and plant growth. It was hoped that resource-rich lignin could play a beneficial role in improving soil structure and promoting crop growth. The phenolic hydroxyl group of lignin was epoxidized and further aminated to load with arbuscular mycorrhizal fungi. The results indicated that amine-modified lignin could effectively load with arbuscular mycorrhizal fungi. The application of arbuscular mycorrhizal fungi-supported aminated lignin to soil aggregate structure improvement greatly reduced the bulk density of soil, and increased the porosity of soil and the content of large granular soil. Compared with unmodified soil, soil bulk density decreased by 73.08%, the porosity of soil increased by 70.43%, and the content of large granular soil increased by 56.38%. Using the improved soil for corn cultivation efficiently increased the biomass of corn. The plant height was increased by 72.16%, the root–shoot ratio was increased by 156.25%, and other indexes were also improved to varying degrees. The experimental method provides an important basis for the effective utilization of lignin materials in agriculture in the future.

## 1. Introduction

Lignin is an important component of plant fiber raw materials, and is a three-dimensional network structure aromatic polymer with abundant resources and a complex structure in nature [1]. During pulping, lignin is generally treated as waste, and the recovery of heat energy through combustion treatment is a great waste of precious lignin resources [2]. According to statistics, China produces over 50 million tons of lignin in papermaking every year [3]. How to efficiently and conveniently achieve high-value conversion and utilization of lignin has always been a focus of research.

**Figure 1:**
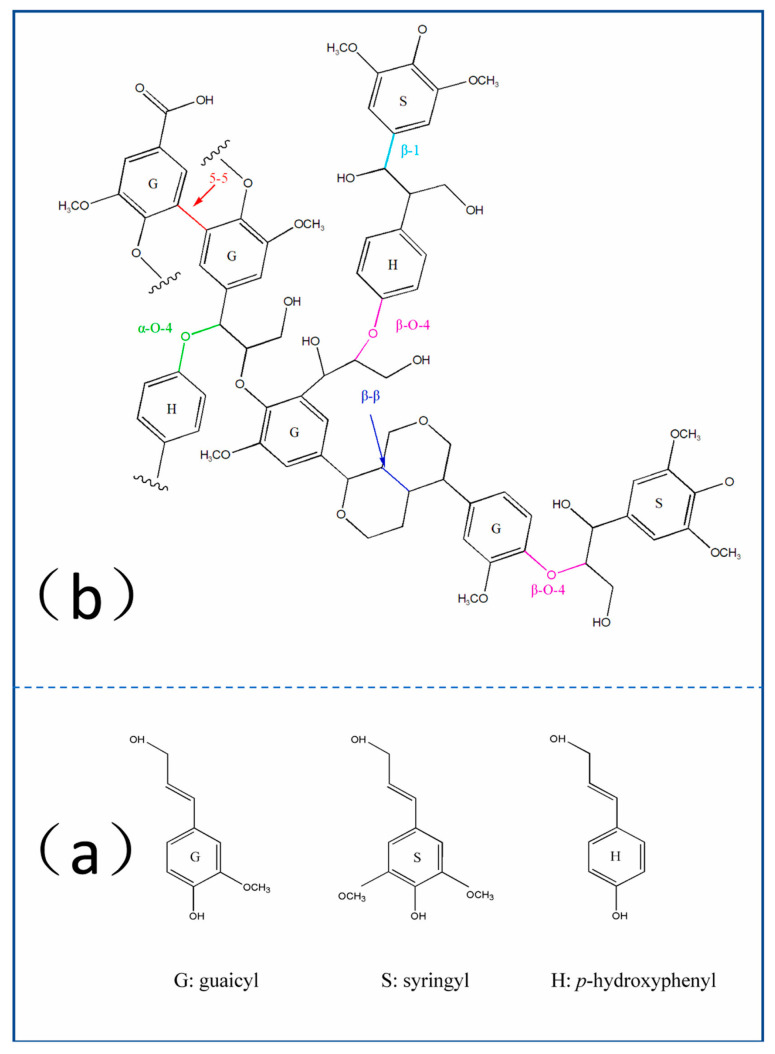
(**a**) Basic structural units of lignin, (**b**) A fragment of lignin structure and its main linkages. Reproduced with permission from ref. [8].

As shown in Figure 1, the basic structural units of lignin, including hydroxyphenyl (H), syringal (S), and guaiacyl (G), are synthesized into polymers through a series of oxidative free radical coupling reactions [4]. In higher plants, lignin chemically connects with cellulose and hemicellulose in the cellulose fiber wall, providing stiffness and strength to the plant structure, as well as resisting the biodegradation of carbohydrates (i.e., enzymatic hydrolysis) and environmental stress [5]. Lignin is considered to play an important role in soil organic carbon cycling and stability [6], and due to its rich active groups, colloidal properties, and structure, lignin can be decomposed by microorganisms into humic acids, thereby improving the structure of soil aggregates, increasing soil permeability, and indirectly affecting microbial activity [7].

Lignin contains a variety of active functional groups, such as phenol hydroxyl, alcohol hydroxyl, methoxy, carboxyl, etc., which have certain binding properties [9]. When applied to soil, lignin can improve soil particle structure, buffer soil acidity and alkalinity, and increase cation exchange capacity [10]. In the acidic soil water environment, the active alcohol hydroxyl group of the lignin breaks, forming positively charged cementation compounds in the soil. Soil colloidal particles are usually finely crushed silico-aluminate crystals, and silicon or aluminum ions located in the center of the lattice are high-priced positive ions, which need to attract the same number of negatively charged ions in order to achieve electrical neutrality [11,12]. When silicon and aluminum ions are replaced by low-priced positive ions, the number of negative charges in the soil colloids is higher than that of positive charges, causing soil colloids to exhibit electronegativity [12]. Under the action of electrostatic force, the positive charge in the lignin can neutralize the negative charge in the soil colloid, thus reducing the thickness of the diffusion double layer [13]. Therefore, based on the theory above, the long polymer chain of lignin can gather soil particles together to form aggregates [14], thereby improving the soil aggregate structure, increasing the soil porosity, and improving the soil permeability. However, when lignin is not modified, due to the existence of carboxyl groups, it still exhibits a high negative charge even under acidic conditions, resulting in electrostatic repulsion between lignin and negatively charged soil particles, which is not conductive to the formation of soil aggregates. The nitrogen atoms in the amine group are positively electric, and the presence of lone pair electrons makes it easy to protonate and form hydrogen bonds. After amination modification, lignin has amine groups on its surface, so that the aminated lignin can be protonated and positively charged under acidic conditions [15], which can strengthen the cementation effect of lignin on soil particles [16]. Thus, aminated lignin can be used to enhance soil structural stability. 

Arbuscular mycorrhizal (AM) fungi are a class of specialized biological nutrients that colonize the root cortex of plants and develop an external mycelium that grows in the soil around plant roots [17]. The basic morphological structure of AM fungi includes five parts [18]: arbuscles, vesicles, internal hyphae, external hyphae, and spores. After successful colonization, AM fungi first secrete a soil protein, glomalin-related soil protein (GRSP) [19], which is a natural organic matter that aggregates smaller than 2 mm into large aggregates (2–5 mm) through binding. Saccharomyces plays an important role in soil structure formation and has a strong positive correlation with the stability of soil aggregate [20]. Finally, the soil particles of various sizes are surrounded by external fungal hyphae through physical entanglement, further aggregating the soil particles and maintaining their stability [21,22]. The improvement of soil aggregation can not only improve the physical properties of soil, such as increasing soil porosity, reducing soil erosion, increasing water infiltration rate, etc., but also reduce the mineralization rate of soil organic matter and nutrient leaching [23]. Morris et al. tracked and localized the soil aggregation process using X-ray fluorescence microscopy and found that AM fungi increased the formation of large aggregates and reduced the decomposition of large and small aggregates. Soil aggregation was the main driving factor for soil stability, confirming that the presence of AM fungi enhanced the mechanical stability of soil [24].

In order to improve the cementation performance of lignin on soil particles, different types of amendments were prepared by aminating primary lignin and loading AM fungi with aminating-modified lignin. The effect of different amendments on soil improvement was evaluated by soil bulk density, porosity, aggregate composition, and stability, as well as the plant height, ground diameter, and biomass of maize plants after a pot experiment. This study provides a theoretical basis and method reference for the high-value utilization of lignin, and also provides a better idea for creating environmentally friendly, economic, and sustainable green agriculture.

## 2. Materials and Methods

### 2.1. Materials

Enzymatic hydrolysis lignin was purchased from Shandong Longlive Bio-Technology Co., Ltd. (Dezhou, China). Corn seed (Zhengdan 958) was obtained from Shandong Jinnong seed industry Co., Ltd. (Liaocheng, China). Hydrochloric acid (HCl) was sourced from Nanjing Chemical Reagent Co., Ltd. (Nanjing, China). Anhydrous ethanol, epichlorohydrin, triethylene tetramine, sodium hydroxide (NaOH), and ninhydrin were purchased from Aladdin Reagent (Shanghai, China). *Rhiaophagus intraradices* was provided by the Institute of Root Biology, Yangtze University (Jingzhou, China). The chemical reagents used above were analytically pure grade.

The soil used in this experiment was collected from the back mountain of Nanjing Forestry University, and the sampling depth was 0~10 cm. After soil collection, large pieces of soil were broken along the grain, and plant roots, sand, and gravel were removed. The treated soil sample was screened for 5 mm and then air dried naturally. The pH of soil, bulk density, porosity, and other parameters were determined with the air-dried samples. Finally, 500 g of air-dried soil was selected for dry screening, and 50 g soil samples were composed of aggregates of different particle sizes according to their proportion for wet screening, and the proportion of aggregates of different particle sizes was calculated according to the method given by Tisdall [25]. 

### 2.2. Methods

The micrographs of all samples were recorded with SEM (Quanta-200, FEI, Shelbyville, KY, USA. Fourier-transform infrared (FTIR) spectra were obtained with an FTIR infrared spectrophotometer operated between 400 and 4000 cm^−1^ (Vertex 80V, Bruker, Karlsruhe, Germany). Zeta potential value was determined by the SZP-06 Zeta potentiometer (Mutek, Grienweg, Neckartailfingen, Germany), pH value was obtained by PHSJ-3J pH (YIZhi Scientific Instrument Co., Ltd., Shanghai, China), and the soil pot experiment was carried out in an intelligent artificial climate chamber (RTOP-5008, Topu Yunnong Technology Co., Ltd., Hangzhou, China).

### 2.3. Analysis of Soil Aggregate Structure 

Soil structure is an important basis for maintaining soil function, supporting animal, plant, and microbial life activities, and good environmental quality. As the foundation of soil structure, soil aggregates promote the formation and evolution of soil structure, so the formation and distribution of aggregates are the focus of soil structure research. At the same time, the pH value of soil is also an essential factor to maintain the stable operation of soil ecological environment. Therefore, in order to understand the structural characteristics of the test soil, the microscopic morphology of the soil was first characterized and observed, and on this basis, the pH value of soil, bulk density, porosity, and aggregate distribution were evaluated.

#### 2.3.1. Determination of the pH Value of Soil

In total, 10 g of air-dried soil was taken into a beaker, and 25 mL of deionized water was added into the beaker, stirring thoroughly with a glass rod for 2 min, and standing for 30 min; then the pH value was determined by a pH meter.

#### 2.3.2. Calculation of Soil Bulk Density

The undamaged original soil was collected using a ring knife with a certain volume (generally 100 cm^3^). After the ring knife was filled with soil samples, the soil samples were then transferred to aluminum boxes of known quality (the mass of the aluminum box was recorded as *M*_1_). Thereafter, the aluminum boxes containing soil were placed in an oven, baked at 105 °C for 4 h, and then cooled in a dryer before being weighed (the mass of the aluminum box containing the soil sample was recorded as *M_2_*, accurate to 0.01 g). The soil bulk density was calculated by the following formula [26],

(1) $$BD=\frac{M_{2}-M_{1}}{V}$$

where: *BD* is the soil bulk density (g·cm^−3^), *M*_1_ is the mass (g) of the empty aluminum box (with cover), *M*_2_ is the mass (g) of the aluminum box (with cover) containing the dried soil sample, and V is the ring cutter volume (cm^3^).

#### 2.3.3. Calculation of Soil Porosity

Soil porosity refers to the percentage of the volume of pores in the soil to the total volume of soil in the natural state. The sampling method described in Section 2.3.2 was used to sample the soil. The calculation formula of soil porosity is as follows [27],

(2) $$P\%=1-\frac{\mathrm{Soil}\;\mathrm{particle}\;\mathrm{density}}{\mathrm{soil}\;\mathrm{density}}\times 100$$

where: soil particle density was calculated using an average value of 2.65 g·cm^−3^.

#### 2.3.4. Soil Water Stability Aggregate Distribution Test

Soil aggregates were determined by a screening method, which including dry screening and wet screening. In the dry screening method, the sieve group (diameter 5.0 mm, 3.0 mm, 2.0 mm, 1.0 mm, 0.5 mm, 0.25 mm) was set from top to bottom according to the sieve hole from small to large. No less than 500 g of air-dried soil was taken and poured on the top layer of the screen group. Next, the sieve group was moved by hand, and the soil samples on each level of the screen were weighed. The percentage content of each level of dry sieve aggregate was calculated. In the wet screening method, according to the percentage content of aggregates at all levels obtained by the dry screening method, the dry sample taken by dry screening was proportively matched to 50 g. The sieve set was set up according to the dry sieve method, and placed on the shock rack of the analyzer in the regiment, putting it into the bucket with water added, starting the machine, and shocking for 30 min. After the machine stopped shocking, the screen group was raised to the water surface, the screen group was split, and the aggregate remaining on the screen was washed into an aluminum box of known quality, heated and dried, weighed, that was, the weight of the water-stable aggregate at all levels, and then the percentage content of the aggregate at all levels was calculated. In this experiment, water-stable aggregate was the main research object.

### 2.4. Aminating Modification of Lignin

#### 2.4.1. Purification of Hydrolysis Lignin

Since the residual sugar and ash in the original lignin would affect the experimental results, an acid-precipitation method was used in this experiment [28]. The enzymatic hydrolysis lignin was dissolved in sodium hydroxide solution in a concentration of 1.67 wt%, stirring to make the lignin dissolved thoroughly and standing for a minute. Then, the suspension was transferred to 1 M HCl with a rubber eyedropper, and the precipitation was washed with deionized water; the purified lignin (PL) was obtained after freeze drying and then was used in the following procedures.

#### 2.4.2. Epoxidation of Lignin

**Scheme 1:**
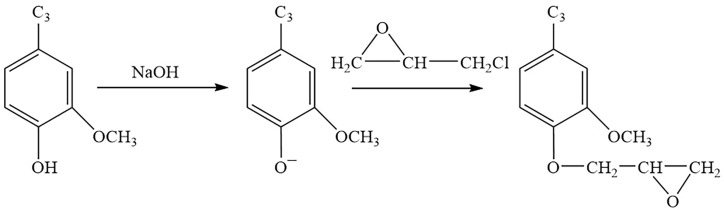
Epoxidation reaction of lignin.

The purified lignin in an amount of 6 g was dissolved in 30 mL sodium hydroxide solution (wt% = 1.67%). Then, epichlorohydrin (molar ratio of phenolic hydroxyl group: epoxy group was 1:9) was dropped in a reaction system. The mixture was heated up to and kept at 80 °C in the oil bath for 3 h under stirring. After the reaction was completed, the reaction mixture was filtered and the residue solid was washed with 95 wt% ethanol and distilled water. The residue solid was dried at 40 °C under vacuum to constant weight and then ground into powder so that the epoxidated lignin (EL) was obtained. The reaction is shown in Scheme 1.

#### 2.4.3. Amination of Epoxy Lignin

**Scheme 2:**
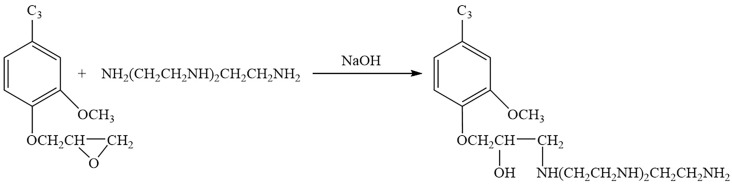
Amination reaction of epoxidated lignin.

The epoxidated lignin powder was dissolved in 1.67 wt% sodium hydroxide solution, and then triethylene tetramine (molar ratio of epoxy groups to amine groups was 1:1, 1:1.5, 1:2, respectively) was dropped into the reaction system. The reaction was kept under 50 °C for 3 h. After the reaction was completed, the residue solid was separated by a filtration process and washed by distilled water, and then dried under vacuum at 40 °C to obtain aminated lignin [29]. In order to distinguish the lignin generated under the above different molar ratios, the abbreviation AL represented the aminated lignin, and the volumes of triethylene tetramine used in the reaction process were taken as the subscripts, which were AL_30_, AL_40_, and AL_50_. The reaction process is shown in Scheme 2.

#### 2.4.4. FTIR Analysis

The infrared spectra of purified lignin, epoxidized lignin, and aminated lignin were determined by Fourier-transform infrared spectrometer. The sample to be tested was dried in an oven at 60 °C for 3 h by the potassium bromide pressing method, and then 200 mg of potassium bromide and 2 mg samples were mixed, ground, and mixed in an agate mortar, and then the pressing mechanism used to make thin slices for testing. The measuring range was 4000–400 cm^−1^, and 32 scans were performed.

#### 2.4.5. Ninhydrin Test

According to the method of Hong et al. [29], 0.2 g ninhydrin was dissolved in 50 mL anhydrous ethanol, and a 5 mL solution was added to a 0.01 g sample. The mixed system was bathed in 80 °C water for 5 min. Finally, the color change of the sample was observed and recorded.

#### 2.4.6. Zeta Potential

A 50 mg sample was evenly dispersed in 5 mL deionized water, and the potential value of the sample was measured by Zeta Sizer Nano Series.

### 2.5. Loading of Arbuscular Mycorrhizal Fungi

AM fungi were loaded by the adsorption method [30]. Briefly, 40 g of fungus sand was taken and soaked in the deionized water and stirred evenly. The prepared aminated lignin was added to the fungal solution, followed by being cultured in a constant temperature oscillator (120 rpm) at 30 °C for 24 h. The obtained products were reserved carefully for following use.

#### FTIR Analysis

The sample preparation and testing methods were the same as those in Section 2.4.4.

### 2.6. Investigate the Influence of Different Samples on Soil Aggregate Structure

In order to investigate the influence of different samples on soil aggregate structure, we selected corn as the experimental object to carry out a pot experiment.

#### 2.6.1. Characterization of Soil Microscopic Morphology

The air-dried soil sample was fully ground with a quartz mortar until the soil was powdered. A 500 mg sample was smeared on the electron microscope tape, and the sample was treated with gold spray, and then the soil morphology was observed under a scanning electron microscope.

#### 2.6.2. Pretreatment of Potting Soil

The collected soil was passed through a 2 mm sieve firstly in order to remove the animal and plant residues and stones, etc., followed by being sterilized in an autoclave at 121 °C for 30 min.

#### 2.6.3. Corn Seed Budding

Forty corn seeds were soaked in distilled water for flotation, and the seeds with full particles were screened. Then, the selected seeds were put into 75% ethanol and stirred continuously with a glass rod for 30 s for surface sterilization. After washing with sterile distilled water 5–6 times, the seeds were placed on Whatman filter paper moistened with distilled water. The filter paper loaded with seeds was placed in a petri dish and germinated in a dark place at room temperature for 3 days. The seeds can be used in pot tests when they produce shoots longer than 1 mm.

#### 2.6.4. Pot Experiment

A total of 1.5 kg of fresh soil was added to each pot, whose height was 20.2 cm and base diameter was 16.3 cm. Different samples of different mass percentages to soil were separately added to the soil (in this study, lignin, aminated lignin, aminated lignin loading with arbuscular mycorrhizal fungi were set as the experimental groups, respectively, and an original soil sample was used as the blank control group). Next, 5 budding corn seeds were planted in each pot. All the potted plants were cultured in an artificial climate incubator, with the temperature set at 25 ± 2 °C, the moderate setting at 70 ± 5% RH, the light intensity at 5500 LX, the light duration at 12 h, and the culture time at 3 months.

#### 2.6.5. Calculation of Soil Bulk Density and Soil Porosity

The soil bulk density and soil porosity were calculated following Equations (1) and (2).

#### 2.6.6. Determination of Apparent Biomass of Maize Plants

In order to directly reflect the effect of samples on soil improvement, the apparent appearance of maize plants was photographed and analyzed by camera and the biomass of maize plants was tested after 3 months. The length from the soil surface to the highest point after the extension of the corn leaves was measured as the plant height, and the diameter of the corn stem at a distance of 10 cm from the ground was measured as the ground diameter. In addition, the dry weight of corn was listed as a test index. Using the surface of the potting soil as a reference frame, the corn plants were cut into roots and ground parts with scissors, washed with clean water, defoliated at 120 °C in the oven for 2 h, dried at 75 °C to constant weight, and weighed with a balance. 

#### 2.6.7. Statistical Analysis

Collected data were analyzed through one-way analysis of variance using Statistical Analysis System (SPSS), version 26, and differences among mean values were processed by the Duncan test. Significance was defined at *p* < 0.05.

## 3. Results and Discussion

### 3.1. Analysis of Soil Aggregate Structure 

As shown in Table 1, the pH of the soil is 5.99, indicating that the tested soil was slightly acidic, which is favorable for maintaining the activity of soil micronutrients [31]. Soil bulk density and porosity are the reflection of soil tightness, which is closely related to soil permeability and nutrient transport [32]. The bulk density value of the tested soil was 1.3 g/cm^3^ and the porosity was 51%. According to the classification standard [33], the soil should belong to the clay soil. Due to the characteristics of small particles and small intergranular pores, clay soil has poor ventilation permeability, high tillage resistance, and poor quality, which is not conducive to the growth and development of plants.

**Figure 2:**
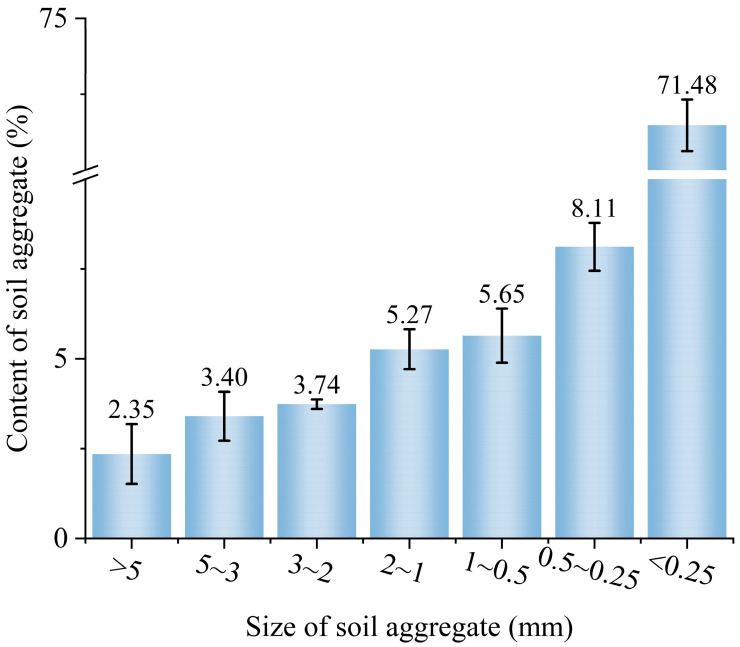
Soil water-stable aggregates.

Previous studies have shown that compared with micro-aggregates, large aggregates contain more organic carbon, nitrogen, and granular organic matter [34,35], and the low content of large aggregates may lead to the lack of organic carbon and other nutrients stored in soil, the decline of soil fertility and cultivability, and the smooth flow of air and water in soil [36]. The distribution diagram of the original soil water-stable aggregates collected in this experiment is shown in Figure 1. The aggregate distribution was dominated by micro-aggregates < 0.25 mm, while the content of water-stable aggregates larger than 0.25 mm was only 28.52%. As illustrated in Figure 2, with the increase in particle size, the content of aggregate decreased step by step. According to the evaluation index of soil water stability proposed by researchers, when the content of water-stable aggregates is between 20 and 30%, the soil belongs to the soil with poor water stability, so it can be judged that the original soil collected in this study is not conducive to the growth and development of plants.

### 3.2. Aminating Modification of Lignin

Due to its colloidal properties and abundant active groups, lignin can improve soil structure when applied to soil. However, the high content of the carboxyl group in lignin makes it show strong electronegativity, which produces electrostatic repulsion on soil particles, which is not conducive to the formation of soil aggregates. The nitrogen atoms in the amine group are positively electric, and the presence of lone pair electrons makes it easy to protonate and form hydrogen bonds [37]. Therefore, the amination modification was introduced into lignin by ring-opening polymerization of epichlorohydrin. Infrared spectrum rendering, the ninhydrin test and the zeta potential test were used to evaluate the modification effect, respectively. 

**Figure 3:**
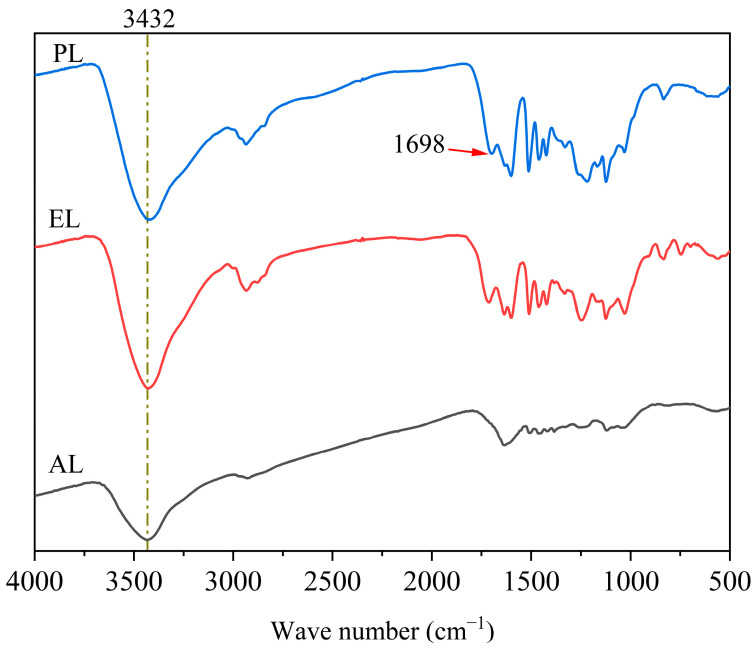
FTIR spectra of purified lignin (PL), epoxidated lignin (EL), and aminated lignin (AL).

As shown in Figure 3, and infrared peaks and their corresponding groups are shown in Table 2. The vibration peaks of the C=C skeleton in the lignin structure (1510 cm^−1^ and 1463 cm^−1^, respectively) were present in the spectra of all three samples, indicating that the lignin structure was not significantly damaged during the modification process. In the infrared spectrum of purified lignin (PL), the peak at 1698 cm^−1^ was attributed to the C=O tensile vibration of carboxyl or carbonyl groups, and the stretching vibration absorption peak of O–H was found at 3432 cm^−1^. In the infrared spectrum of epoxidated lignin (EL), new peaks appeared at 1125 cm^−1^ and 1029 cm^−1^ that could be judged as the stretching vibration peak of epoxy ring C–O–C. Meanwhile, the open chain stretching vibration peak of C–O at 1245 cm^−1^ and the characteristic absorption peak of C–Cl bond at 748 cm^−1^ further indicated that part of epichlorohydrin did not close but existed in a free state after ring opening. The above results showed that the epichlorohydrin graft was successful. Focusing on aminated lignin (AL), the bending vibration in the N–H plane of the aliphatic secondary amine at 1633 cm^−1^ was stronger than the spectrum of sample EL. The N–H stretching vibration absorption peak on the aminated lignin was 3432 cm^−1^, and the peak type here was wider than that of sample PL, indicating that the peak in sample AL was the superposition of the O–H stretching vibration absorption peak and the N–H stretching vibration absorption peak. In addition, the flexural vibration peak of the single substituted epoxide at 832 cm^−1^ disappeared. The change in these characteristic peaks indicated the success of the amination modification of lignin [29].

**Figure 4:**
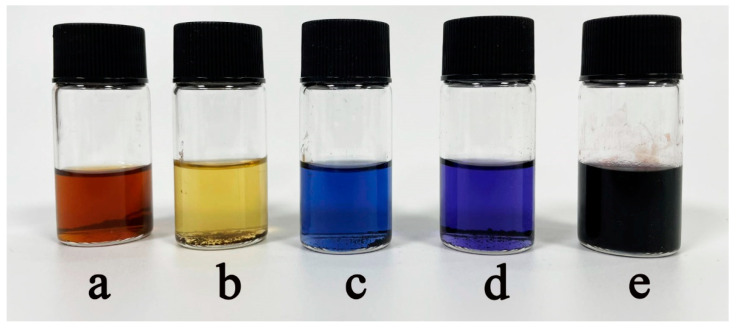
Color reactions of PL (**a**), EL (**b**), (**c**–**e**) aminated lignin sample AL_30_, AL_40_, and AL_50_.

The ninhydrin test is a visual measurement reflecting the presence of amine groups in the substance by reacting ninhydrin with amine groups in the substance to form complexes with specific colors [29]. When there is no amine group in the substance, the reaction system will be yellow, but when there is an amine group in the substance, the reaction system will appear blue, purple, or blue-purple, and the greater the content of amine groups in the reaction system, the darker the color. In Figure 4a,b, the solution is yellow, indicating that no amine groups were present in the reaction system. In Figure 4c,d,e, the solution gradually changes from blue to blue-purple, indicating that the lignin was successfully grafted with amine groups, the content of amine groups in the system showed a trend of gradual increase, and AL_50_ contained the most amine groups.

**Figure 5:**
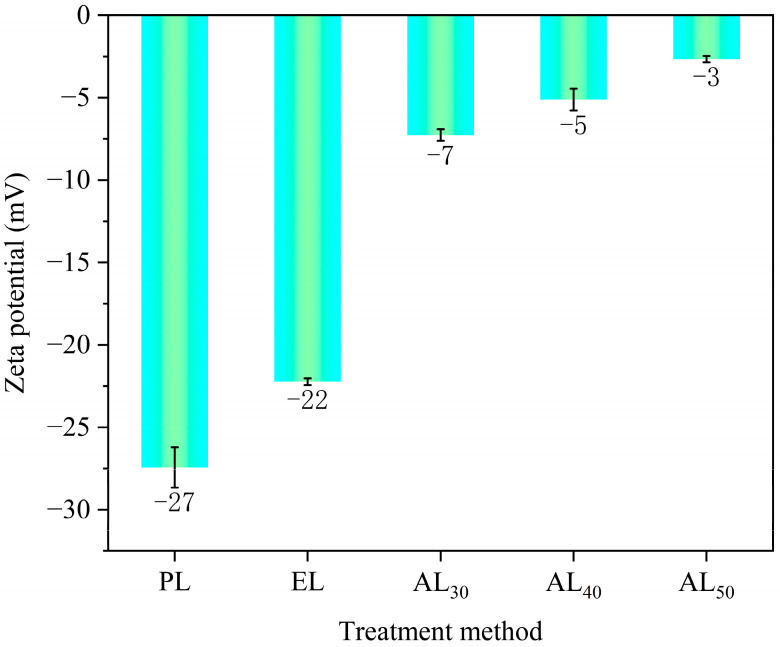
Zeta potential of purified lignin (PL), epoxidated lignin (EL), aminated lignin (AL_30_, AL_40_, AL_50_).

Different chemical structures and electronegative functional groups will directly affect the type and amount of charge on the surface of particles [38]. Zeta potential values were also measured to demonstrate the effect of modification on the surface charge of the lignin. As shown in Figure 5, lignin precipitated by hydrochloric acid dissolved by sodium hydroxide has a potential of −27 mV due to the presence of hydroxyl and carboxyl functional groups in its molecular structure. After epichlorohydrin grafting, the potential increased to −22 mV because part of the phenol hydroxyl group was consumed. Further, after grafting the polyamine compound, the potential of AL_50_ increased to −3 mV due to the introduction of amine groups. 

### 3.3. Immobilization of Arbuscular Mycorrhizal Fungi

In order to achieve the ideal improvement effect, it is necessary to add a lot of amendments to the soil, but excessive amendments will cause a burden to the soil environment, such as plugging the soil pores [39]. Arbuscular mycorrhiza (AM) is the most common symbiotic relationship between plants and microorganisms. AM fungi are found in most soil environments and are able to provide a range of important ecological services, including improved uptake and utilization of plant nutrients, increased plant stress and tolerance, and improved soil structure and fertility [40], thereby increasing crop productivity. Therefore, aminated lignin was used as a carrier to support arbuscular mycorrhizal fungi (AMF) by the adsorption method. The load effect was evaluated by infrared spectroscopy.

**Figure 6:**
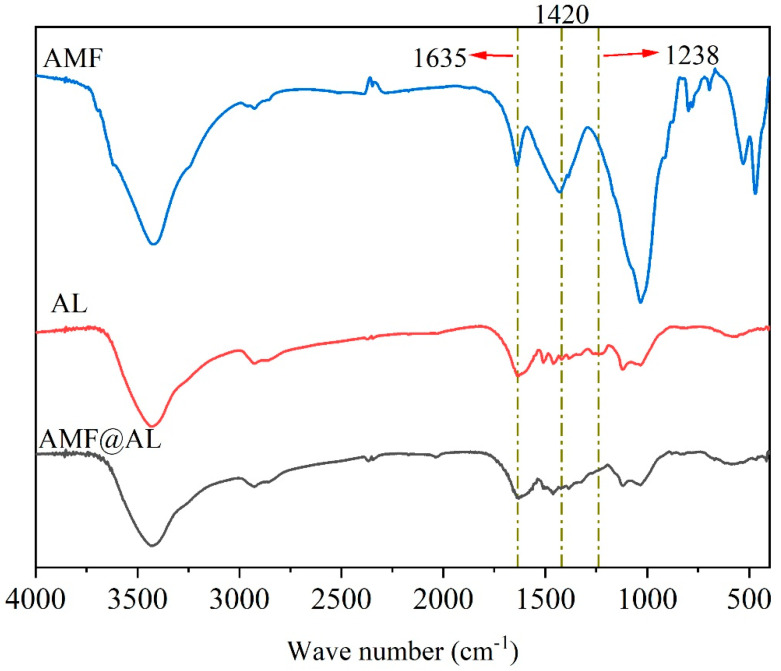
FTIR spectra of arbuscular mycorrhizal fungi (AMF), aminated lignin (AL), and aminated lignin loading arbuscular mycorrhizal fungi (AMF@AL).

The infrared spectra of arbuscular mycorrhizal fungi, aminated lignin, and aminated lignin supported by arbuscular mycorrhizal fungi is shown in Figure 6, and infrared peaks and their corresponding groups are shown in Table 3. In the absorption band of AMF, the C=O stretching vibration of amide I was present at 1635 cm^−1^, and the C–N stretching vibration of amide III was present at 1420 cm^−1^. In the AL spectrum, the bending vibration of the carboxyl group (–COOH) was found at 1238 cm^−1^. By comparing the absorption peaks of AL and AMF@AL, it was found that the peak at 1238 cm^−1^ disappeared, while the oxygen energy on acids, aldehydes, or ketones on lignin formed hydrogen bonds with the hydrogen of amide groups on the cell surface [41,42]. By comparing the absorption peaks of AMF and AMF@AL, it was found that the peak at 1420 cm^−1^ in the spectrum of AMF@AL decreased. This was related to the conformational changes in proteins caused by the involvement of adsorption on the cell surface [41]. The changes in the absorption peaks indicated that AMF was successfully loaded on AL through hydrogen bonding.

### 3.4. Investigation of the Influence of Different Samples on Soil Aggregate Structure

**Figure 7:**
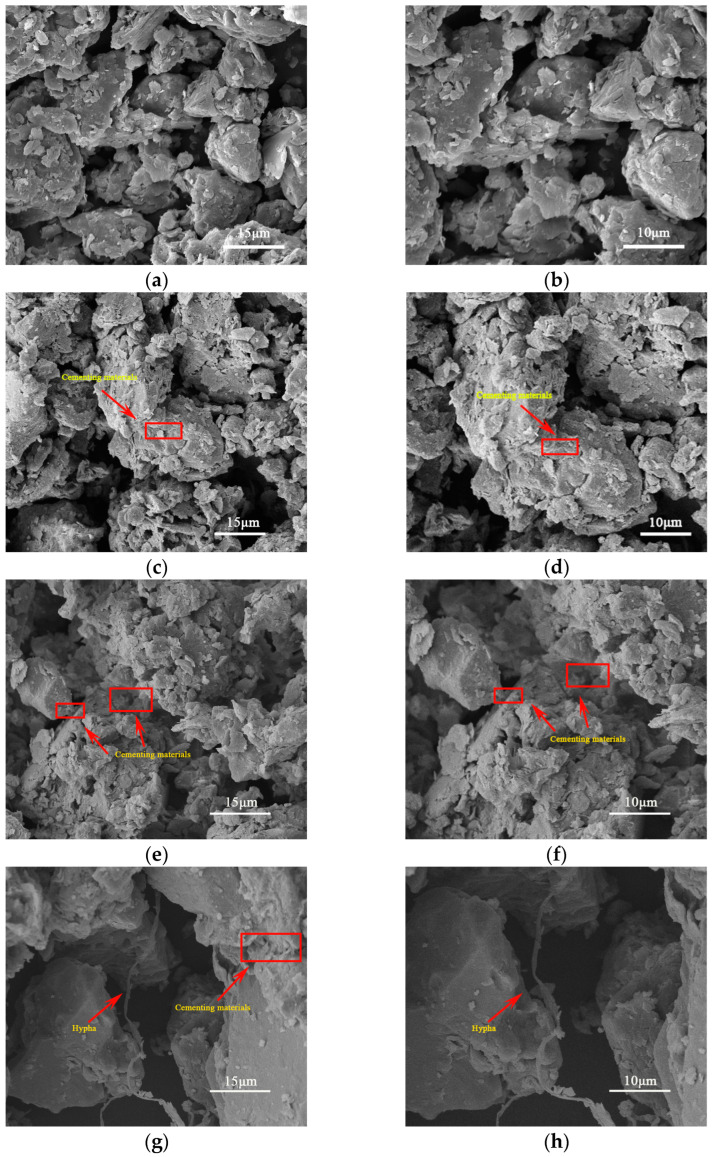
The SEM images of original soil (**a**,**b**), soil treated by purified lignin (**c**,**d**), soil treated by aminated lignin (**e**,**f**), and soil treated by AMF-loaded aminated lignin (**g**,**h**).

In order to directly evaluate the effect of improved materials on soil structure, the micro-morphology of improved and unimproved soil was observed and analyzed, and on this basis, the indexes of bulk density, porosity, and aggregate composition were measured. As shown in Figure 7a,b, there were a lot of pores in the unimproved soil as a whole, and clear boundaries existed between the solid particles. In addition, the surface of an individual soil particle was smooth and angular, and the particles were filled with many smaller ones. After being improved by purified lignin, soil particles were coated and bonded by the cementing materials formed by lignin conversion, and the pores between particles were partially filled (Figure 7c,d). When soil was improved by aminated lignin, as illustrated in Figure 7e,f, the distance between particles became closer, the boundaries became blurred, and many particles stacked on top of each other because cementing materials filled the pores between the soil particles and bound the particles to each other. After AMF-loaded aminated lignin was applied to soil improvement, in addition to the cementing materials between particles, hypha could also be observed to attach to the surface of soil particles and extend to both sides to entangle soil particles (Figure 7g,h).

**Figure 8:**
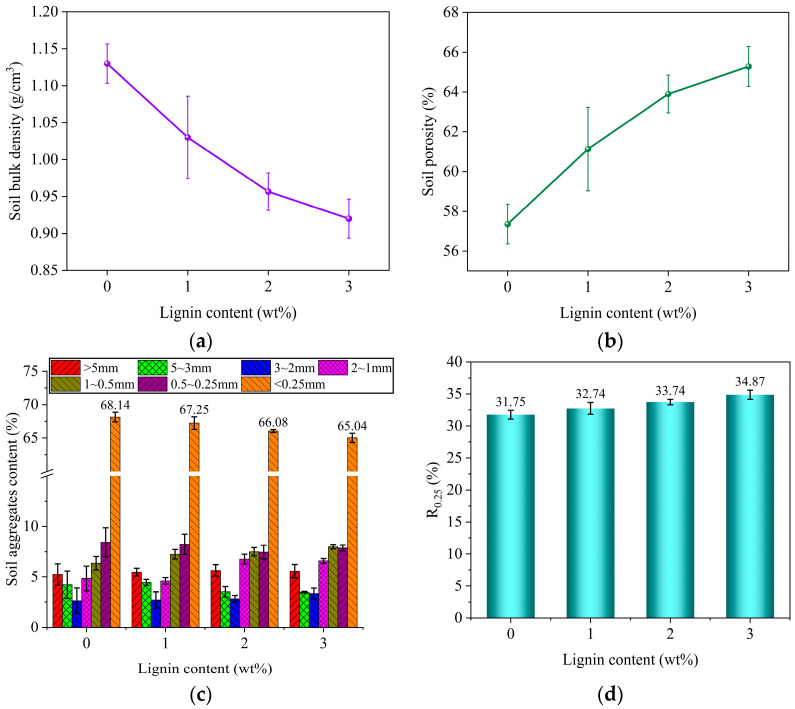
Soil bulk density (**a**), soil porosity (**b**), soil water-stable aggregates content (**c**), and aggregates content larger than 0.25 mm (**d**): treated by original lignin.

Through the analysis of original soil characteristics, it could be easily found that the original soil sample is not conducive to the growth and development of plants. What is exciting is that the experimental results showed that the addition of lignin could effectively improve the soil aggregate structure, and the introduction of amine groups and AM fungi could further optimize the improvement effect. With the increase in the volume of the addition of lignin, the soil bulk density decreased first and then leveled off (Figure 8a), and the soil porosity gradually increased and then leveled off (Figure 8b). Specifically, when the volume of the addition of lignin was 3 wt%, the improvement effect of the structure was the best. Compared with the control group, the soil bulk density decreased by 29.23%, while the porosity increased by 28.01%. As shown in Figure 8c, aggregates smaller than 0.25 mm also decreased gradually and then tended to be flat, while aggregates larger than 0.25 mm increased first and then tended to be flat. When lignin content was 3 wt%, the total aggregate larger than 0.25 mm increased by 9.83% compared with the group without lignin addition (Figure 8d).

In addition, the mean weight diameter value of soil at this time reached 0.85 (in Table 4), which was at the level of water-stable soil. However, because the unmodified lignin itself was difficult to dissociate in the experimental soil, the aggregate of the soil was still dominated by small particle size aggregates.

**Figure 9:**
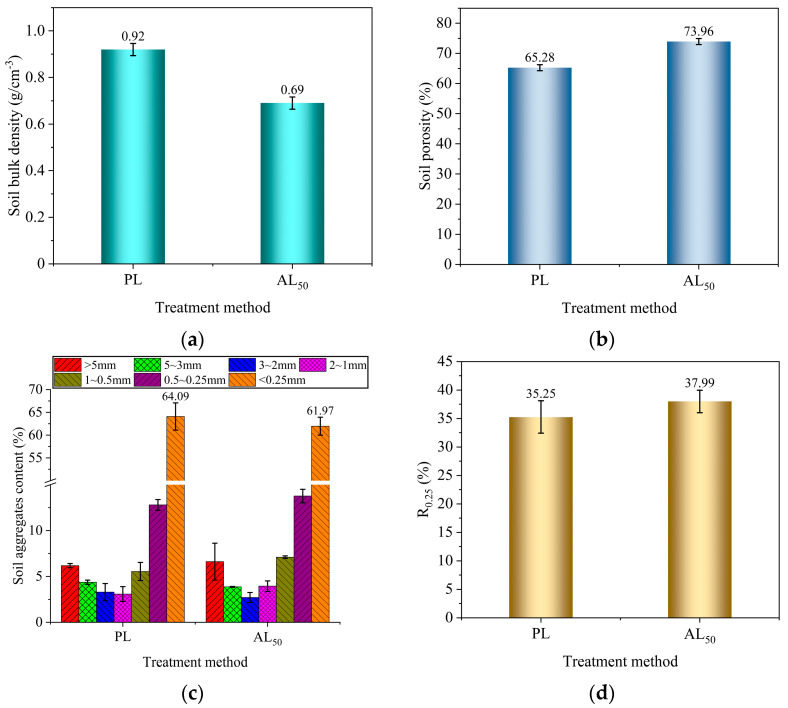
Soil bulk density (**a**), soil porosity (**b**), soil water−stable aggregates content (**c**), and aggregates content larger than 0.25 mm (**d**): treated by purified lignin (PL) and aminated lignin (AL_50_).

Based on the experimental results above, the samples of unmodified lignin and aminated lignin AL_50_ were selected for a pot experiment next, and the materials were added to the soil at 3 wt% of the soil mass, respectively. Compared with soil treated by unmodified lignin, the bulk density of potting soil treated with amine-modified lignin decreased by about 25% (Figure 9a). We suspect there could be a number of reasons for this decline. Firstly, the positivity of amine groups induced electrostatic attraction to the electronegative soil particles [43], which strengthened the cementation of lignin and deepened the degree of soil agglomeration. Secondly, the amine-modified lignin can be used as nitrogen fertilizer, which can be absorbed and utilized by plant roots when applied in soil to promote root growth [44]. In addition, roots can wrap soil particles, and root secretions can bond soil particles together, thus promoting the formation of soil aggregates [45]. The results of porosity further proved our hypothesis. Compared with the unmodified lignin, the porosity of potting soil treated with amine-modified lignin increased by 13.29% (Figure 9b). As shown in Figure 9c,d, compared with the original lignin, the content of aggregates smaller than 0.25 mm in the soil treated with aminated lignin was further reduced, and the content of aggregates larger than 0.25 mm increased by 7.77%.

As shown in Table 5, compared with soil treated by original lignin, the MWD of soil treated by aminated lignin increased by 5.88%, and the value of GMD increased slightly by 2.90%.

**Figure 10:**
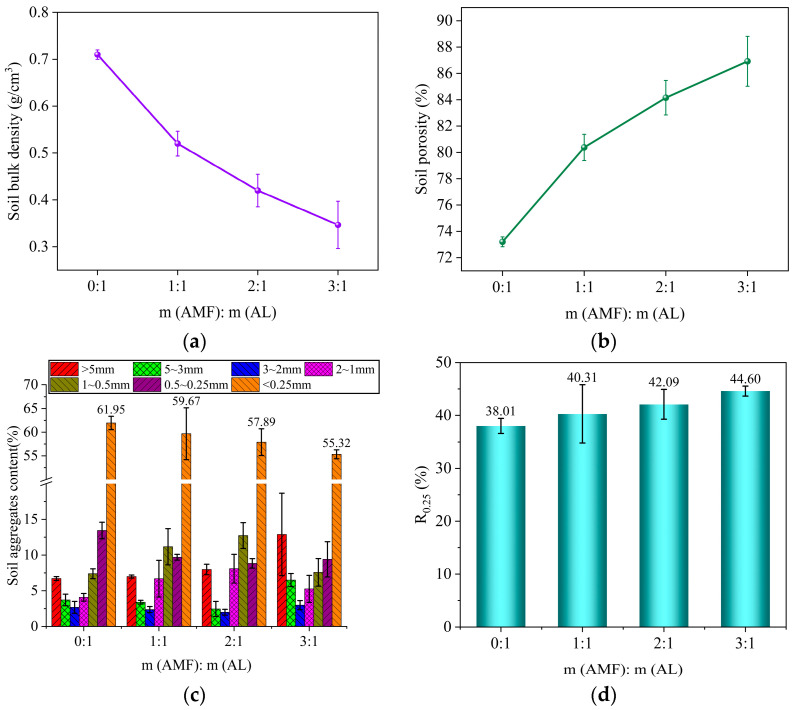
Soil bulk density (**a**), soil porosity (**b**), soil water-stable aggregates content (**c**), and aggregates content larger than 0.25 mm (**d**): treated by aminated lignin loading AMF.

It is worth noting that existing studies have shown that although the introduction of a positive charge can strengthen the attractive force on soil particles, this force is not stable [46]. If the particle size of soil aggregates increases, the acting distance will be increased and the acting force will be weakened. The addition of arbuscular mycorrhizal fungi can make up for the insufficient effect of aminating lignin. It can bind and wrap soil particles with mycelium, narrow the distance between soil particles [47], and provide conditions for the electrostatic attraction of lignin amine. At the same time, arbuscular mycorrhizal fungi can secrete a soil protein as an adhesive, which acts synergistically with amine lignin to adsorb fine soil particles. Compared with ammoniated lignin alone, after loading of arbuscular mycorrhizal fungi, the soil bulk density decreased by 49.28% (Figure 10a) and the soil porosity increased by 17.52% (Figure 10b). Except for the differences mentioned above, the content of aggregates smaller than 0.25 mm declined, while the content of those larger than 0.25 mm increased, as shown in Figure 10c,d. All these changes indicated a substantial improvement in soil aggregate structure.

As can be seen in Table 6, the mean weight diameter of soil was 1.29, and the geometric mean diameter value was 0.79. These changes revealed that the soil structure was amended as well.

### 3.5. Investigation of the Influence of Different Samples on Plant Biomass

**Figure 11:**
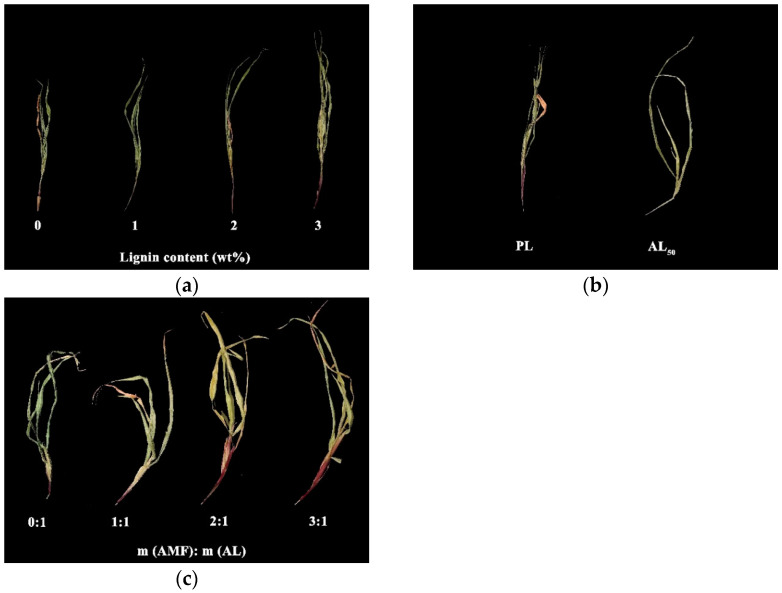
Plant growing in lignin-treated soil (**a**), plant growing in aminated lignin-treated soil (**b**), plant growing in aminated lignin loading AMF-treated soil (**c**).

In order to evaluate the improvement effect of various materials on soil structure, the apparent morphology and biomass of maize plants were measured and analyzed. Figure 10a,b,c shows the effects of different improved materials on the apparent morphology of maize plants. Lignin, aminated lignin, and AMF-loaded lignin treatment promoted plant growth. The growth of the plants treated with lignin was better than that of the groups treated without modification (Figure 11a). This might be because lignin, in addition to improving the soil aggregate structure, was also a growth regulator in itself, promoting plant growth [48]. As shown in Figure 11b, the growth of plants treated with aminated lignin was better than that treated with lignin because the introduction of amines not only enhanced the promotion of soil agglomeration [49] but also increased the content of nitrogen fertilizer in soil. Finally, the stem coarseness and plant length of plants treated with AMF-loaded aminated lignin were higher than those treated with aminated lignin (Figure 11c), indicating that the addition of AMF promoted the absorption and utilization of soil nutrients by plants, and also reflected the further optimization of soil structure.

The experimental results showed that when arbuscular mycorrhizal fungi supported by aminating lignin were used to improve soil, the optimization degree of soil structure would gradually increase and then become flat as the mass ratio between arbuscular mycorrhizal fungi and aminating lignin increased. Therefore, in this experiment, the biomass of soil plants with the best effect without adding improved materials and with adding various amendments was selected for comparison, and the results are shown in Table 7. The plant biomass of improved soil was higher than that of unimproved soil. It can be seen from the data in the table that the maize plant biomass of AL_50_-improved soil was better than that of PL-improved soil, while AMF@AL-improved soil continued to be optimized on the basis of AL_50_. When the mass ratio of AMF to AL was 3:1, the plant height was increased by 72.16%, the root–shoot ratio was increased by 156.25%, and other indexes were also improved to varying degrees. This also indicated that the soil aggregate structure had been greatly improved, which also provided more space for the life activities of plant roots, facilitating the extension of the root system and the absorption and utilization of nutrients.

## 4. Conclusions 

In this study, we successfully modified lignin by amination, and realized the highly efficient loading of arbuscular mycorrhizal fungi by the adsorption method. The application of arbuscular mycorrhizal fungi-supported aminated lignin to soil aggregate structure improvement can greatly reduce the soil bulk density of soil, and increase the porosity of soil and the content of large granular soil. Compared with unmodified soil, soil bulk density decreased by 73.08%, the porosity of soil increased by 70.43%, and the content of large granular soil increased by 56.38%. Using the improved soil for corn cultivation can efficiently increase the biomass of corn. Compared with unimproved soil, the plant height was increased by 72.16%, the root–shoot ratio was increased by 156.25%, and other indexes were also improved to varying degrees. The results showed that the modified materials could improve the soil aggregate structure and promote the respiration of plants and the absorption of nutrients. The experimental method provides an important basis for the effective utilization of lignin materials in agriculture in the future.

## Figures and Tables

**Table 1:** Basic characteristics of soil.

pH Value	Soil Bulk Density (g/cm^3^)	Soil Porosity (%)
5.99	1.3	51

**Table 2:** Infrared peaks and their corresponding groups of FTIR spectra of purified lignin (PL), epoxidated lignin (EL), and aminated lignin (AL).

Peak/cm^−1^	Corresponding Group
1463, 1510	C=C
1698	C=O
3432	O–H, N–H
1125, 1029	C–O–C
1245	C–O
748	C–Cl
1633	N–H

**Table 3:** Infrared peaks and their corresponding groups of FTIR spectra of arbuscular mycorrhizal fungi (AMF), aminated lignin (AL), and aminated lignin loading arbuscular mycorrhizal fungi (AMF@AL).

Peak/cm^−1^	Corresponding Group
1635	C=O
1420	C-N
1238	-COOH

**Table 4:** Mean weight diameter and geometric mean diameter of soil amended by original lignin.

Lignin Content (wt%)	Mean Weight Diameter (mm)	Geometric Mean Diameter (mm)
0	0.76 ± 0.029 ^a^	0.68 ± 0.020 ^a^
1	0.82 ± 0.019 ^b^	0.69 ± 0.005 ^ab^
2	0.84 ± 0.011 ^b^	0.70 ± 0.003 ^b^
3	0.85 ± 0.022 ^b^	0.71 ± 0.012 ^b^

Different superscript characters (a,b) within the same column indicate significant differences between the samples (*p* < 0.05).

**Table 5:** Mean weight diameter and geometric mean diameter of soil treated by original and aminated lignin.

Treatment Method	Mean Weight Diameter (mm)	Geometric Mean Diameter (mm)
PL	0.85 ± 0.030	0.69 ± 0.021
AL_50_	0.90 ± 0.013	0.71 ± 0.011

**Table 6:** Mean weight diameter and geometric mean diameter of soil amended by aminated lignin loading with AMF.

m (AMF)–m (AL) (wt%)	Mean Weight Diameter (mm)	Geometric Mean Diameter (mm)
0:1	0.87 ± 0.030 ^a^	0.70 ± 0.005 ^a^
1:1	0.91 ± 0.063 ^a^	0.72 ± 0.026 ^a^
2:1	0.94 ± 0.037 ^a^	0.73 ± 0.016 ^a^
3:1	1.29 ± 0.260 ^b^	0.79 ± 0.043 ^b^

Different superscript characters (a,b) within the same column indicate significant differences between the samples (*p* < 0.05).

**Table 7:** Comparison of plant biomass of maize growing in untreated soil and soil treated by purified lignin (PL), aminated lignin (AL_50_), and aminated lignin loading AMF (AMF@AL).

Treatment Method	Stem Diameter (mm)	Plant Height (cm)	Root Dry Weight (g)	Shoot Dry Weight (g)	Root–Shoot Ratio
Untreated	1.63 ± 0.122 ^a^	25.50 ± 0.764 ^a^	0.04 ± 0.005 ^a^	0.23 ± 0.014 ^a^	0.16 ± 0.012 ^a^
PL	2.03 ± 0.065 ^a^	30.10 ± 1.258 ^b^	0.07 ± 0.003 ^b^	0.29 ± 0.002 ^b^	0.23 ± 0.011 ^b^
AL_50_	2.64 ± 0.025 ^b^	36.30 ± 1.041 ^c^	0.10 ± 0.004 ^c^	0.33 ± 0.015 ^c^	0.30 ± 0.009 ^c^
AMF@AL_(0:1)_	2.68 ± 0.112 ^b^	36.90 ± 1.000 ^c^	0.11 ± 0.007 ^c^	0.35 ± 0.033 ^cd^	0.31 ± 0.050 ^c^
AMF@AL_(1:1)_	2.85 ± 0.350 ^bc^	38.10 ± 2.466 ^c^	0.12 ± 0.014 ^d^	0.37 ± 0.025 ^de^	0.33 ± 0.023 ^c^
AMF@AL_(2:1)_	3.09 ± 0.377 ^cd^	40.60 ± 2.255 ^c^	0.14 ± 0.002 ^e^	0.42 ± 0.024 ^e^	0.35 ± 0.021 ^cd^
AMF@AL_(3:1)_	3.47 ± 0.239 ^d^	43.90 ± 3.122 ^d^	0.25 ± 0.009 ^f^	0.61 ± 0.011 ^f^	0.41 ± 0.009 ^d^

Different superscript characters (a–f) within the same column indicate significant differences between the samples (*p* < 0.05).

## Data Availability

The original contributions presented in the study are included in the article, further inquiries can be directed to the corresponding author.
